# Simultaneous and quantitative monitoring of co-cultured *Pseudomonas aeruginosa* and *Staphylococcus aureus* with antibiotics on a diffusometric platform

**DOI:** 10.1038/srep46336

**Published:** 2017-04-12

**Authors:** Chih-Yao Chung, Jhih-Cheng Wang, Han-Sheng Chuang

**Affiliations:** 1 Department of Biomedical Engineering, National Cheng Kung University, Tainan, Taiwan; 2 Department of Urology, Chimei Medical Center, Tainan, Taiwan; 3 Medical Device Innovation Center, National Cheng Kung University, Tainan, Taiwan

## Abstract

Successful treatments against bacterial infections depend on antimicrobial susceptibility testing (AST). However, conventional AST requires more than 24 h to obtain an outcome, thereby contributing to high patient mortality. An antibiotic therapy based on experiences is therefore necessary for saving lives and escalating the emergence of multidrug-resistant pathogens. Accordingly, a fast and effective drug screen is necessary for the appropriate administration of antibiotics. The mixed pathogenic nature of infectious diseases emphasizes the need to develop an assay system for polymicrobial infections. On this basis, we present a novel technique for simultaneous and quantitative monitoring of co-cultured microorganisms by coupling optical diffusometry with bead-based immunoassays. This simple integration simultaneously achieves a rapid AST analysis for two pathogens. Triple color particles were simultaneously recorded and subsequently analyzed by functionalizing different fluorescent color particles with dissimilar pathogen-specific antibodies. Results suggested that the effect of the antibiotic, gentamicin, on co-cultured *Pseudomonas aeruginosa* and *Staphylococcus aureus* was effectively distinguished by the proposed technique. This study revealed a multiplexed and time-saving (within 2 h) platform with a small sample volume (~0.5 μL) and a low initial bacterial count (50 CFU per droplet, ~10^5^ CFU/mL) for continuously monitoring the growth of co-cultured microorganisms. This technique provides insights into timely therapies against polymicrobial diseases in the near future.

Various antibiotics are constantly developed since the first antibiotic, penicillin, was discovered by Fleming in 1928. However, with a global increase in antibiotic resistance of bacterial infections^1^,^2^, antimicrobial susceptibility testing (AST) is a pivotal step in seeking an effective medication for infections^3^ The current gold standard of AST is broth microdilution, which usually consumes over 24 h and requires at least 10^7^ CFU/mL of microorganisms for analysis^4^,^5^,^6^. Molecular techniques, such as multi-PCR^5^, for detecting resistant genes are usually time-saving but cannot determine the minimum inhibitory concentration (MIC) and unknown antibiotic-resistant genes^1^. An empirical antibiotic therapy is therefore necessary. However, this kind of treatment causes poor patient response and sprawling of multidrug-resistant pathogens^1^,^7^. A timely and efficient screening for drug susceptibility is needed to overcome these problems^1^,^3^,^7^.

In clinical practices, the polymicrobial nature of many diseases or infections was confirmed and gained attention from the community^8^,^9^,^10^. Most diseases are verified as monomicrobial through culture isolation techniques^9^. However, with the advancement of methodologies, diseases recognized as polymicrobial infections are increasing^9^. For example, *Pseudomonas aeruginosa* and *Staphylococcus aureus* are usually found in chronic wound infections or cystic fibrosis^10^,^11^,^12^. This synergistic coexistence is expected to enhance virulence, increase antimicrobial resistance, and prolong recovery time for hosts^10^,^11^,^12^. Developing AST for these infections is important to address the complexity of polymicrobial infections^8^. Consequently, rapid, efficient, and simultaneous monitoring of AST for co-cultured bacteria can save lives under the threat of sepsis and prevent the proliferation of superbugs^8^.

Numerous techniques based on morphological analysis^13^, fluorescence intensity^14^,^15^, asynchronous magnetic bead rotation^16^,^17^, dielectrophoresis^18^, Raman-enhanced spectroscopy^19^,^20^, atomic force microscopy^21^ and surface plasmon resonance imaging^22^ are developed to improve AST. These techniques use direct quantification factors (including counting by image analysis^13^, fluorescence intensity^14^,^15^ and bacterial proliferation volume^16^) and indirect factors (including morphology^13^,^18^ medium viscosity^16^, bacteria-disrupted secretion^19^,^20^ and bacterial fluctuations originating from metabolism^21^,^22^) to efficiently determine the antibiotic susceptibility profiles of bacteria. These techniques require a timeframe of 0.5–4 h to a complete an AST. This timeframe is faster than that of their conventional counterparts. However, their clinical use is limited by various issues, such as inconsistency with the conventional method, limited scope of applications, sophisticated fabrication procedures, and requirements of state-of-art equipment. Furthermore, only a few of the mentioned techniques can deal with polymicrobial infections.

In our previous study, we successfully established an optical diffusometric platform for achieving rapid AST and quantifying the growth of microorganisms depending on Brownian motion^23^. Brownian motion which describes the random movement of suspending particles is self-driven and subjected to ambient temperature, liquid viscosity, and particle size. The random displacement, *x*, of a particle caused by Brownian motion is associated with the time interval, ∆*t*, and diffusion coefficient, *D*. The relation was described by Langevin^24^ and Einstein^25^ as


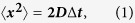


where


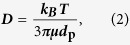


where *k*_*B*_ is the Boltzmann constant, *T* is the absolute temperature of fluid, *μ* is the viscosity of fluid, and *d*_p_ is the particle diameter. At constant temperature and liquid viscosity, the diffusion coefficient is simply a function of the particle diameter. Techniques based on measuring Brownian motion are used to detect M13 viruses^26^ and C-reactive protein^27^. Their result shows that the Brownian motion of particles decreases when the particles are bound with target analytes^26^,^27^. In our experiment, we replaced viruses with bacteria. Prticles were conjugated with corresponding antibodies depending on our target bacteria (Fig. 1A). We proposed that when the bacteria grow and attach to particles as a probe, the Brownian motion of the particles declines in response to the increased equivalent particle diameter (Fig. 1B). When the bacteria are sensitive to the antibiotic, the declining trend stops (Fig. 1B)^23^. By using serial dilution of an antibiotic in several chips, we determined the MIC of the antibiotic against a certain bacterium according to the aforementioned criteria. We revealed that simultaneous AST is achieved within 2 h using *P. aeruginosa* and *S. aureus* co-cultured and combined with pathogen-specific antibodies and fluorescent particles. The sample volume was only 0.5 μL, and the initial bacterial number was as low as 10^5^ CFU/mL. The droplet loaded on the chip was continuously monitored over a long timescale (at least 2 h). From our perspective, more applications relevant to quantifying coexisting microorganisms can be performed in the same fashion.

## Results

### Optical Diffusometry Fabrication and Images Splitting

**Figure 2:**
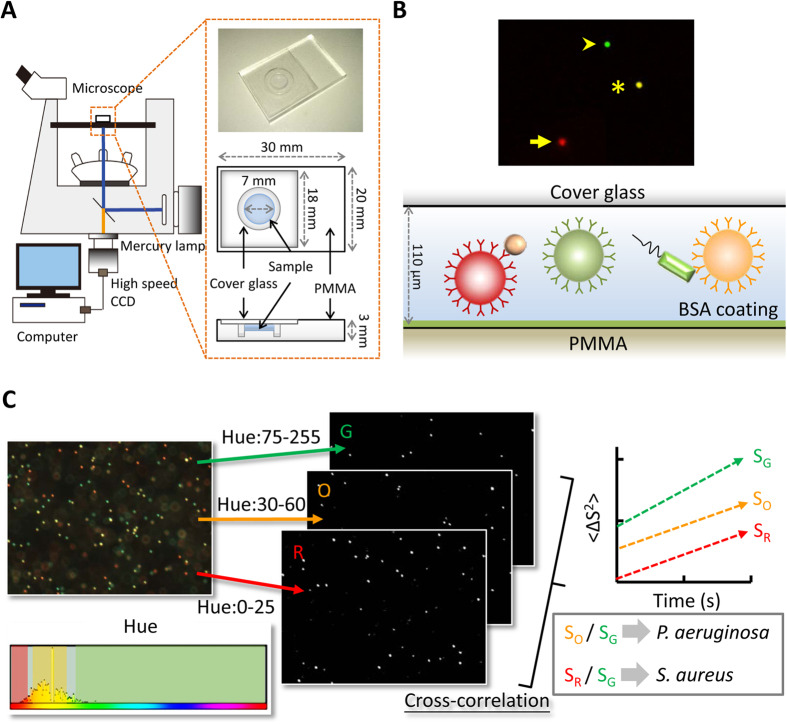
Optical diffusometric platform. (**A**) Optical diffusometry and design of PMMA chip. (**B**) Illustration of the multi-pathogenic detection with triple-fluorescent particles. Green, orange, and red fluorescent particles were functionalized with anti-TNF-α Ab, anti-*P. aeruginosa* Ab, and anti-*S. aureus* Ab, respectively. Anti-*P. aeruginosa* Ab- and anti-*S. aureus* Ab-functionalized particles were used to simultaneously detect the bacteria, and the anti-TNF-α Ab-functionalized particles served as a probe to reflect the environmental conditions. (**C**) Workflow of Brownian motion determination for multi-fluorescent color image sets. Triple or dual color particle image sets were split using ImageJ by sampling hue range, which yielded image sets of each fluorescent color. The cross-correlation algorithm was used to extract the corresponding diffusivity values. The diffusivity values of the anti-*P. aeruginosa* Ab- and anti-*S. aureus* Ab-functionalized particles (S_O_ and S_R_) were then divided by the diffusivity of the anti-TNF-α Ab-functionalized particles (S_G_) to remove the measurement variations that resulted from different environmental factors or background noise.

Optical diffusometric platform was composed of an epifluorescent microscope, a digital camera, and a chip for sample loading (Fig. 2A). Considering that bead-based immunoassays take advantage of a large reaction surface area, a small sample quantity, and the simultaneous determination of multiple pathogens^28^,^29^, we further expanded the platform to conduct simultaneous and quantitative monitoring of the growth of co-cultured bacteria. Pathogen-specific antibodies and fluorescent particles were combined to probe different pathogens (Fig. 2B). Triple or dual color particle images were split using ImageJ by sampling hue range, which yielded image sets of each fluorescent color (Fig. 2C). Split image sets were then analyzed by the cross-correlation algorithm to evaluate their corresponding diffusivity values (Supplementary Fig. S1). The relative diffusivity values were obtained by dividing the diffusivity of pathogen-specific Ab-functionalized particles by that of the reference particles, which removed the measurement variations that resulted from different environmental factors or background noise (Fig. 2C).

### Efficacy and specificity of bead-based immunoassay

**Figure 3:**
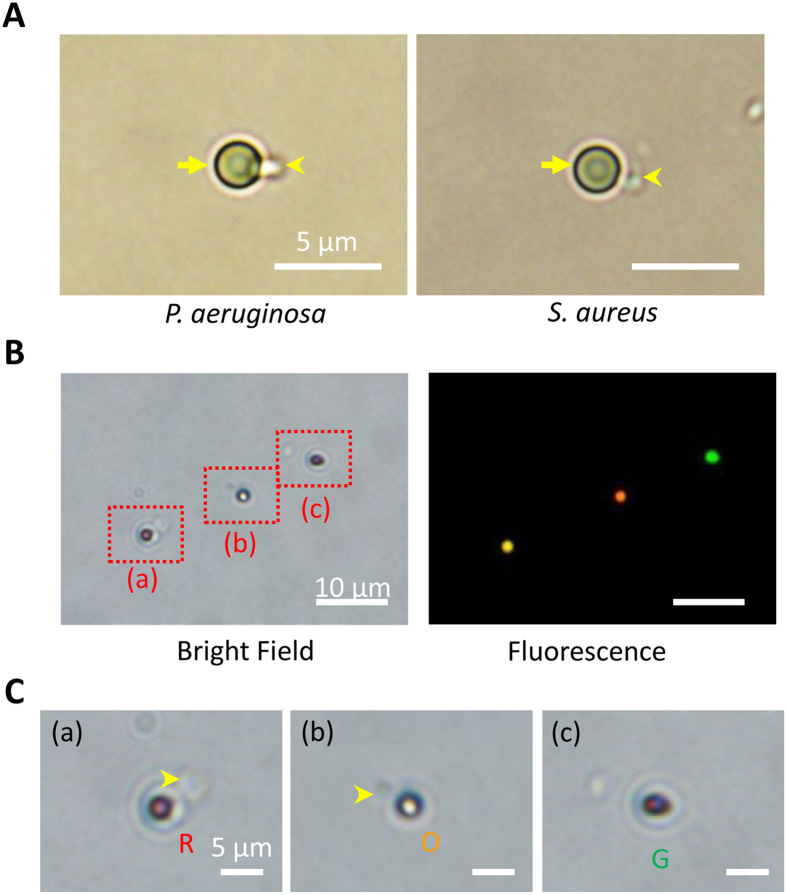
Bindings between microorganisms and particles. (**A**) Optical microscopic image of *P. aeruginosa* (left panel) and *S. aureus* (right panel) attached to an Ab-functionalized particle. The scale bar is 5 μm. (**B**) Fluorescence microscopic images of simultaneous multi-pathogenic detection with triple-fluorescent particles (bright field, left panel; fluorescent field, right panel). The scale bar is 10 μm. (**C**) Close views of red rectangles labeled as (a), (b), and (c) in Fig. 3B. (a) *S. aureus* attached to anti-*S. aureus* Ab-functionalized red fluorescent particles (R in red). (b) *P. aeruginosa* attached to anti-*P. aeruginosa* Ab-functionalized orange fluorescent particles (O in orange). (c) Anti-TNF-α Ab-functionalized green fluorescent particles free from bacterial binding (G in green). The scale bar is 5 μm.

Six types of particles, namely, non-modified, carboxylate-modified, amine-modified, anti-*P. aeruginosa* Ab-functionalized, anti-*S. aureus* Ab-functionalized, and anti-TNF-α Ab-functionalized particles incubated with *P. aeruginosa* or *S. aureus* were investigated to evaluate the binding efficacy and specificity (Table 1) under an optical microscope. Ab-functionalized particles were prepared by EDC/NHS crosslinking (Supplementary Fig. S2). Mixed *P. aeruginosa* and *S. aureus* were incubated with a blend of green, orange, and red particles functionalized with anti-TNF-α Ab, anti-*P. aeruginosa* Ab, and anti-*S. aureus* Ab, respectively. The anti-TNF-α Ab-functionalized particles were free from non-specific binding of bacteria and thus served as a reference to reflect the environmental fluctuations. For anti-*P. aeruginosa* Ab- and anti-*S. aureus* Ab-functionalized particles, high efficacies of specific binding with their corresponding bacteria were observed after incubation for 1 h (Fig. 3A).

The strategy for simultaneous detection of a mixed bacterial sample was identified by functionalizing different color particles with various antibodies. In this study, green fluorescent particles were coated with anti-TNF-α Ab and served as a reference probe. Orange fluorescent particles coated with anti-*P. aeruginosa* Ab and red fluorescent particles coated with anti-*S. aureus* Ab were used to capture *P. aeruginosa* and *S. aureus* bacteria, respectively. The image of bright field provided evidence of the binding between the mixed microorganisms and their corresponding particles (green: environmental reference; orange: *P. aeruginosa*; and red: *S. aureus*) (Fig. 3B,C, and Supplementary Fig. S3). Particular bacteria are selectively caught on functionalized particles under bacterium-mixed condition. This result proved that the simultaneous detection of multipathogens is achieved by immunoassays.

### Simultaneous detection of multiple bacteria based on bead-based immunosensing

**Figure 4:**
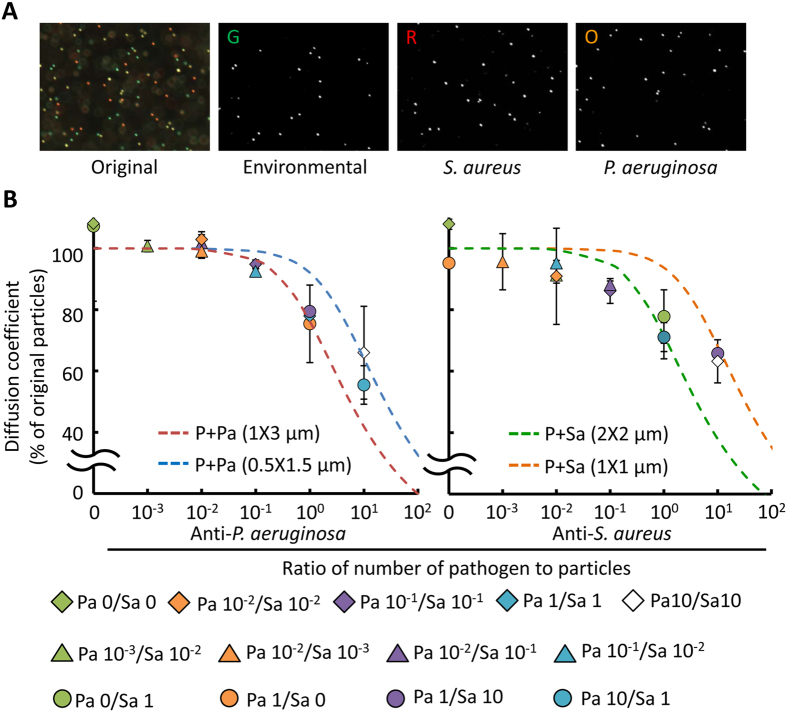
Simultaneous multi-pathogenic detection and quantification with functionalized triple-fluorescent particles. (**A**) Triple or dual color particle image sets split using ImageJ by sampling hue range, which yielded image sets of each fluorescent color. (**B**) Mean diffusivity values of functionalized particles (Anti-*P. aeruginosa* Ab, left; Anti-*S. aureus* Ab, right) attached with their corresponding bacteria mixed at different concentrations. The dotted lines are the predicted curves of 2-μm particles with respect to different bacterial concentrations and sizes according to the equivalent diameter theory. P means particles, Pa means *P. aeruginosa*, Sa means *S. aureus*, and the number behind Pa and Sa means the ratio of bacteria to their corresponding particles.

A phenomenon similar to our prior work^23^ was observed in particles bound with dead or non-motile bacteria, such as *S. aureus*; more bacteria always result in lower diffusivity because of the larger equivalent particle diameter (Supplementary Fig. S4A,B). Subsequently, the diffusivity values of the two functionalized particles at different *S. aureus* concentrations were simultaneously recorded with two different color particles composed of equal amounts of anti-*S. aureus* and anti-TNF-α Ab-functionalized particles. A correlation peak of the image pairs was calculated by fast Fourier transform. Two-dimensional Gaussian curve was applied to define the width of the correlation peak, which is theoretically accosiated with diffusivity. We sequentially rotated each correlation domain by 90° for a series of consecutive correlation peaks to achieve an ensemble average^23^. After the image processing, the diffusivity values of anti-*S. aureus* Ab-functionalized particles divided by those of anti-TNF-α Ab-functionalized particles were compared using the equivalent diameter theoretical model. The results showed favorable agreement with the theoretical curves of 2-μm particles (Supplementary Fig. S5). Two bacteria, *P. aeruginosa* and *S. aureus*, were also measured at different concentrations in a mixed sample by using triple color particles composed of equal amounts of anti-*P. aeruginosa* Ab- (orange), anti-*S. aureus* Ab- (red), and anti-TNF-α (green) Ab-functionalized particles. UV-sterilized *P. aeruginosa* and *S. aureus* were incubated with the functionalized particles at varied ratios (Table 2). After image splitting and processing (Fig. 4A), the relative diffusivity values of orange and red particles were obtained by dividing their measured diffusivity values by that of green particles to avoid the influences of environmental factors and background noise. The relative diffusivity declined with the increased bacterial density (diamonds, triangles and circles; Fig. 4B). A theoretical model based on the equivalent volume diameter^30^ was also used to predict the interactions between Ab-functionalized particles and dead bacteria (dotted lines, Fig. 4B). The bacteria sizes of *P. aeruginosa* and *S. aureus* were estimated to be 0.5–1 × 1.5–3 and 1–2 × 1–2, respectively. The experimental result agrees with the theoretical prediction. The agreement confirms the feasibility of the diffusion-based measurement technique.

### Rapid antimicrobial susceptibility testing for multiple pathogens

**Figure 5:**
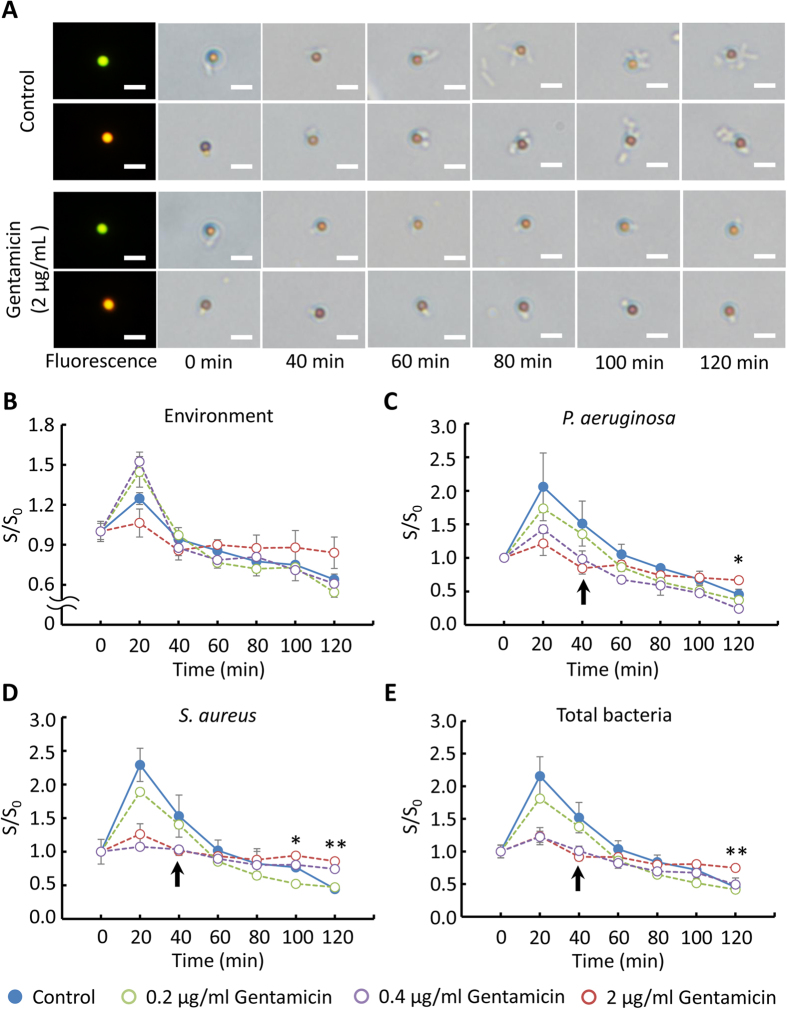
AST evaluation of co-cultured *P. aeruginosa* and *S. aureus* with gentamicin. (**A**) Microscopic images of bacterium-particle complexes. (*P. aeruginosa*, orange fluorescent; *S. aureus*, red fluorescent) with gentamicin (0, 0.2, 0.4, and 2 μg/mL) in a time course. The scale bar is 5 μm. (**B**) Temporal diffusivity changes in anti-TNF-α Ab-functionalized green fluorescent particles. The particles served as a probe to sense the changes in environmental conditions. (**C**) Temporal diffusivity changes in anti-*P. aeruginosa* Ab-functionalized particles with gentamicin (0, 0.2, 0.4, and 2 μg/mL). (**D**) Temporal diffusivity changes in anti-*S. aureus* Ab-functionalized particles with gentamicin (0, 0.2, 0.4, and 2 μg/mL). (**E**) Average temporal diffusivity changes in anti-*P. aeruginosa* Ab- and anti-*S. aureus* Ab-functionalized particles attached with their corresponding bacteria in TSB with gentamicin (0, 0.2, 0.4, and 2 μg/mL). The arrows in (**C**), (**D**), and (**E**) indicate when the bacteria stop growing. S is the slope of regression lines and S_0_ is the regression line slope of particles measured at 0 min. *p < 0.05, and **p < 0.01.

For each AST measurement, the chip was flipped over every 2 min and the recording was performed every 20 min with a 10X objective in a total timespan of 2 h. The technique was validated by the preliminary experimental results and thus was applied to an AST for multiple pathogens. After incubating the bacteria and their corresponding Ab-functionalized particles at a ratio of 1:1, bacterium-binding particles were mixed with gentamicin in TSB medium. For continuously long-term monitoring particle diffusivity (at least 2 h), sedimentation disturbs the diffusometry-based AST even though the density of particles was close to water (ρ = 1.05 g/cm^3^). In addition, adhesion also occurred when the particles were near the chip walls. Sample droplets loaded in the chip coated with or without BSA were flipped at rate of 0, 1 and 2 min^−1^ over 2 h to avoid sedimentation. Numbers of particles in the middle and near-wall planes were then calculated. Results showed that flipping a chip with BSA coating at rate of 1 and 2 min^−1^ effectively avoids particle sedimentation on the near-wall plate (Supplementary Fig. S6). Therefore, the chip loaded with a sample droplet was flipped over every 2 min and continuously monitored every 20 min over a timeframe of 2 h. Without gentamicin (control), the co-cultured bacteria, *P. aeruginosa* and *S. aureus*, continuously reproduced and attached to their corresponding functionalized particles. However, the bacterial growth was drastically suppressed in the presence of 2 μg/mL gentamicin (Fig. 5A). On the diffusometric platform, the diffusivity changes in anti-TNF-α Ab-functionalized particles, an environmental reference, increased in the first 20 min both in the control group and in all the experimental groups. The diffusivity of the control group and the groups of 0.2 and 0.4 μg/mL gentamicin constantly decreased to about 60% of their values at time 0. By contrast, the diffusivity change in the group of 2 μg/mL gentamicin slightly decreased to 85.8% of its initial diffusivity value in the first 60 min and then no change was observed afterwards (Fig. 5B). Given that the anti-TNF-α Ab-functionalized particles were clear of bacterial binding, the diffusivity change in the particles was affected only by bacterial motility and medium viscosity.

Simultaneous and quantitative monitoring of co-cultured *P. aeruginosa* and *S. aureus* with or without MIC gentamicin showed similar results as our previous data in the *P. aeruginosa* mono-cultured condition. In the mono-culture scenario, the diffusivity changes of the particles attached to *P. aeruginosa* in the control group and in the group of 0.2 μg/mL gentamicin all increased in the first 20 min, followed by a constant decrease to about 25% of their initial diffusivity values (Supplementary Fig. S7A). In the current co-cultured condition, however, the diffusivity of the anti-*P. aeruginosa* Ab-functionalized particles in the control group and in the groups of 0.2 and 0.4 μg/mL gentamicin both increased in the first 20 min and then constantly decreased to about 40% of their values at time 0. The declining diffusivity over time indicated failure of the antibiotic on the bacteria. By contrast, the diffusivity of the particles with 2 μg/mL gentamicin slightly decreased to 84.6% of their initial diffusivity values in the first 60 min and afterwards showed no change (Fig. 5C). The trend that the diffusivity ceased declining and maintained a constant over time indicated successful inhibition of the antibiotic on the bacteria. The diffusivity of anti-*S. aureus* Ab-functionalized particles attached to *S. aureus* with 0, 0.2, and 2 μg/mL gentamicin showed the same trend as their anti-*P. aeruginosa* Ab-functionalized particle counterparts except the group of 0.4 μg/mL gentamicin (Fig. 5D). Surprisingly, the CLSI suggested MIC, 0.2 μg/mL gentamicin, failed to inhibit *S. aureus* in the co-cultured condition. In a *S. aureus* mono-culture condition, the diffusivity changes in the functionalized particles of the control and 0.1 μg/mL cefapime groups only displayed a constant decline, whereas the diffusivity of the particles with 1 and 4 μg/mL cefapime showed no change (Supplementary Fig. S7B). Based on the standard broth microdilution, we also tested the effect of gentamicin, which suppressed the bacterial growth at 2 μg/mL (Supplementary Fig. S8). Interestingly, a selected concentration of gentamicin (0.4 μg/mL) showed a different trend that inhibited *S. aureus* but *P. aeruginosa*. In this case, the diffusivity of anti-*P. aeruginosa* Ab-functionalized particles declined with time (Fig. 5C), whereas that of anti-*S. aureus* Ab-functionalized particles remained unchanged (Fig. 5D). This difference was not distinguished by broth microdilution (Supplementary Fig. S8). In addition, this result suggested that a higher concentration to inhibiting the growth of co-cultured bacteria was needed, which is consistent with the prior study^15^. We further averaged the diffusivity values of the anti-*P. aeruginosa* Ab- and anti-*S. aureus* Ab-functionalized particles to better understand the overall growth of the co-cultured bacteria with gentamicin (Fig. 5E). The result showed significant differences between groups at 120 min according to the statistical analysis based on ANOVA.

## Discussion

Our previous study showed that the diffusometry-enabled AST was convenient and reliable^23^. On this basis, we further expanded the technique to AST for co-cultured bacteria by drawing on the features of bead-based immunoassays. The incubation time and binding efficacy for particle–bacterium binding in the present study were consistent with those of the bead-based immunoassay in previous studies^16^,^26^,^31^. Although non-specific bindings remain unavoidable without a special surface modification^32^, this result proved that the simultaneous detection of multipathogens is achieved through immunoassays. Particle aggregation occasionally occurred when particles get a higher chance to encounter the same bacteria because of inhomogeneous dispersion. Statistically, the degree of particle aggregation escalated with the concentration of bacteria. In other words, more bacteria could cause more particle aggregates. Although the aggregation was an unfavorable factor in the study, this trend, in part, contributed to lowering the diffusivity. In addition, the cross-correlation algorithm is a measure of statistical analysis and is thus capable of mitigating the bias resulted from the aggregation. As a result, our overall measurements were not significantly interfered with the particle aggregation. For the diffusivity changes of particles, particles bound with motile bacteria, *P. aeruginosa*, showed larger movement area than that of free particles in our previous work^23^. Other studies also stated that active micro-swimmers, such as algae, alter the particle motion^33^,^34^,^35^. By contrast, the diffusivity of particles proportionally decreased according to the number of dead or non-motile bacteria attached (Supplementary Fig. S4A,B)^23^. The experimental data generally showed favorable agreement with equivalent diameter theory even in the mixed bacterial sample (Fig. 4B). Thus, although different types of bacteria were used, the theoretical curves provide useful predicting information on the sensitivity and limit of detection of our method. On the other hand, ellipsoidal and peanut-like colloids, which are non-spherical particles, displayed biased diffusion with a slight deviation from the predicted diffusive motion because of the coupling effect of translation and rotation^36^,^37^. Accordingly, the deviation of our present experiments is attributed to the size differences and orientations of the bacteria attached to the particles^16^,^36^,^37^. Nevertheless, the cross-correlation algorithm only measured the width of the ensemble correlation peak; hence, the biased diffusion caused by the non-spherical shape is negligible^23^. We further mitigated the biased diffusion by progressively rotating each by 90° and summing the correlation peaks. Different from other studies that use single particles to estimate the change of analytes^16^,^27^, our technique featured an ensemble behavior of a population of particles, yielding an average activity of the overall microorganism. In clinic, our method is not easily misled by a small fraction of data and accurately reflects the overall response of microorganisms to antibiotics.

In the proposed rapid AST technique, our diffusometric platform achieves simultaneous and quantitative monitoring of the growth of co-cultured bacteria by quantifying diffusivity, which was subjected to the viscosity and equivalent diameter change resulting from the bacterial growth and binding. In this study, the variations in diffusivity were affected by several factors, including particle sizes^26^,^27^, particle shapes^36^,^37^ bacterial motility^33^,^34^,^35^ and medium viscosity^38^,^39^. In general, Brownian motion is inversely proportional to the equivalent particle diameter if the bacteria are dead or nonmotile^38^,^39^. Conversely, motile bacteria escalate the diffusivity of particles through collision^33^,^34^,^35^. During bacterial proliferation, the medium viscosity^38^,^39^, equivalent diameter of particles, and bacterial motility increased^33^,^34^,^35^. When the proliferating bacteria in culture broth exceeds a threshold, the increased medium viscosity and particle diameter account for the majority movement of particle^23^ and thereby result in an evident drop in the particle diffusivity. Therefore, the escalation of diffusivity of anti-*P. aeruginosa* Ab and anti-*S. aureus* Ab-functionalized particles (Fig. 5C–E) in the first 20 min resulted from the motility of *P. aeruginosa*. However, with the proliferation of the bacteria, the effect from increased particle diameter and medium viscosity gradually became governing (Fig. 5B). By contrast, the mild decline in diffusivity of the particle–bacterium complexes with 2 μg/mL gentamicin was attributed to the bactericidal effect of gentamicin, which destroyed the motility of *P. aeruginosa*^23^. In the group of 0.4 μg/mL gentamicin, which can inhibit *S. aureus* but not *P. aeruginosa*, the diffusivity change of bacterium corresponding particles distinguishes the different responses of the bacteria (Fig. 5C,D), which cannot be achieved in broth microdilution (Supplementary Fig. S8). Therefore, the diffusivity in this study was an indicator of the summarized effects of the aforementioned factors and reflected the actions of antibiotic on bacteria. Moreover, the diffusivity change in particles in response to motility loss of pathogens with drug treatments can be applied to drug screens in parasitic protozoa diseases.

In our previous study, we defined that susceptibility occurs when the diffusivity changes of the particle–bacterium complexes show a “level” trend; otherwise, the bacteria were resistant^23^. The bacteria in a control group were regarded resistant, whereas the bacteria in the presence of 2 μg/mL gentamicin were susceptible. Notably, the co-existence of *P. aeruginosa* and *S. aureus* in chronic wounds or cystic fibrosis is usually highly resistant to antibiotics^10^,^11^,^12^. The co-culturing of *P. aeruginosa* and *S. aureus* also showed a slower growth rate than individual culturing^12^. The enhanced tolerance is attributed to the host-derived and bacterium-derived matrix components, which require synergistic interactions between bacteria and *in vivo* surrounding environment. These findings explain the antibiotic susceptibility result in our current *in vitro* co-cultured system. The diffusivity changes in pathogen-specific-functionalized particles in the presence of 2 μg/mL gentamicin slightly increased to 121.3–126.2% of their initial values in the first 20 min, which was undetected in our previous study. Moreover, the diffusivity values of pathogen-specific-functionalized particles in the control group after incubation for 120 min were about 40% of their initial values. These values were higher than the values (23.3–28.6%) in our previous study^23^, revealing a slower growth rate than individual bacterial culture (Fig. 5C–E). The results generally suggested that our diffusometric platform is a useful tool to reflect the synergistic interactions among co-cultured microorganisms.

Based on our previous findings that the diffusivity change of particles bound with bacteria can be a sensitive indicator of the quantity of microorganisms, we further developed a diffusometric platform for simultaneous monitoring of co-cultured microorganisms. An AST of co-cultured *P. aeruginosa* and *S. aureus* with gentamicin was quickly conducted within 2 h by analyzing the temporal diffusivity changes in triple-fluorescent particles with different pathogen-specific antibodies. In addition to a small sample volume (~0.5 μL) requirement, a low initial bacterial concentration (10^5^ CFU/mL), high sensitivity (one bacterium on single particles), and simple fabrication, the study herein presents a novel technique that features multiplexed and rapid AST (within 2 h) for continuously monitoring the growth of polymicrobial infection pathogens over a relatively long timescale (at least 2 h). However, different AST outcomes were observed between the *in vivo* and our *in vitro* environments. The discrepancy requires extra effort for investigation in the near future. We expect that the proposed technique will be applied in the research of polymicrobial infections. Our ongoing project of prototype construction that monitors several chips in a row will achieve clinical application of this multiplexed and rapid AST in the near future.

## Methods

### Reagents

Green (L4530, Ex/Em: 360/420), orange (L9529, Ex/Em: 520/540), and red (L3030, Ex/Em: 575/610) fluorescent carboxylate-/amine-modified polystyrene particles (*d*_p_ = 2 μm), 1-ethyl-3-(3-dimethylaminopropyl) carbodiimide (EDC), N-hydroxysuccinimide (NHS), 2-(n-morpholino)-ethanesulfonic acid (MES), bovine serum albumin (BSA), and gentamicin were obtained from Sigma-Aldrich (St. Louis, MO, USA). The particles were used as probes to catch the bacteria in a sample suspension. Gentamicin, an antibiotic for AST, was mixed with bacteria to achieve a final concentration of 2 μg/mL. Anti-*P. aeruginosa* polyclonal antibody (Ab, ab67905), anti-*S. aureus* polyclonal Ab (ab20920), and anti-TNF-α monoclonal Ab (ab9348) were acquired from Abcam (Cambridge, MA, USA). Tryptic soy broth (TSB) was obtained from BD (East Rutherford, NJ, USA). Polymethylmethacrylate (PMMA) was purchased from Sunmei (Tainan, Taiwan).

### Bacterial culture

*P. aeruginosa* (ATCC 27853), a motile gram-negative rod (0.5–1 × 1.5–3 μm), and *S. aureus* (ATCC 23360), a non-motile gram-positive coccus (1–2 × 1–2 μm), were provided by Dr. H. C. Chang of the Department of Biomedical Engineering at Nation Cheng Kung University in Taiwan. *P. aeruginosa* and *S. aureus* were cultured in an incubating shaker at 37 °C in TSB for 12–16 h before use.

### Bead-based immunoassay

Specific bacteria in fluids were captured by bead-based immunoassays. Three different fluorescent polystyrene particles were functionalized with three different pathogen-specific antibodies through EDC/NHS chemistry. EDC (10 mg/mL) and NHS (10 mg/mL) were used to activate the carboxyl groups on the polystyrene particles at a mole ratio of 1:400:1200 for 15 min. The EDC/NHS-activated particles were then incubated for 4 h at 4 °C with the antibodies, and the final volumetric concentration was 0.625% v/v (Supplementary Fig. S2)^23^.

### Bacterial-binding efficacy

Six conditioned particles, namely, non-modified, carboxylate-modified, amine-modified, anti-*P. aeruginosa* Ab-functionalized, anti-*S. aureus* Ab-functionalized, and anti-TNF-α Ab-functionalized particles, were separately incubated with *P. aeruginosa* or *S. aureus* (10^8^ CFU/mL) to understand the efficacy and specificity of bacterium–particle binding. Their binding rates were then measured under an optical microscope. Mixed *P. aeruginosa* and *S. aureus* (10^8^ CFU/mL of each) were incubated with a blend of green, orange, and red particles functionalized with anti-TNF-α Ab, anti-*P. aeruginosa* Ab, and anti-*S. aureus* Ab, respectively (each was 0.0125%, 2.8 × 10^7^ beads/mL), to further verify the feasibility of multi-pathogenic detection with three different color particles. Anti-TNF-α Ab-functionalized particles where bacteria did not bound were used as the negative control. After incubation for 1 h, bacterium–particle complexes were examined under an optical microscope.

### Optical diffusometric platform

In this study, an optical diffusometric platform was built by integrating a fluorescent microscope (IX71, Olympus), a high-speed camera (Flea^®^3 USB 3.0 Cameras, Point Grey) and a PMMA chip (30 mm × 20 mm × 3 mm) (Fig. 2A)^23^,^40^. The observation area was isolated by a circular groove, ensuring that the drop forms a hocky-pocket shape, to define the geometric dimension of a sample drop on the chip. The circular groove on the chip was created by a computer assisted milling machine (EGX-400, Reload DGA). A suspension containing a mixture of bacteria and modified particles was pipetted on the chip and covered with a cover glass (Fig. 2B)^23^,^39^,^40^. The depth of the drop was defined by a spacer of 110 μm. Two fluorescent particles functionalized with two antibodies, anti-*P. aeruginosa* Ab and anti-*S. aureus* Ab, served as probes to detect the existence of corresponding bacteria in the environment. The image plane was focused in the middle depth of the chip to avoid hindered diffusion near the boundaries. A series of particle images was recorded with a 10X objective at a frame rate of 10 Hz for 20 s^23^.

Considering the density difference between particles and water (*ρ*_*p*_/*ρ*_*w*_ = 1.05), sedimentation was further assessed. Assuming particles with a radius of 1 μm were suspended in a water solution (viscosity *μ* ≃ 1 cP), the Stokes sedimentation velocity (*ν*_*S*_ = 2Δ*ρ* *gR*^2^*/9μ*) was estimated to be 1.15 × 10^−1^ μm/s. Given that the particle images were recorded for 20 s and the depth of correlation of the 10X objective was 31.7 μm, the sedimentation distance (2.3 μm) during the measurement was thus negligible^41^. The coefficient of horizontal hindered diffusion in the middle plane was also calculated to evaluate the interferences from the bottom and top walls^40^:


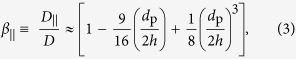


where *h* is the distance between particle and wall, and *D* and *D*_||_ represent the bulk diffusion and the component of the hindered diffusion parallel to the wall, respectively. The high-order terms in Eq. (3) were neglected. With *d*_p_ = 2 μm and *h* = 55 μm (the measurement plane was focused in the middle of the chip), the hindered diffusion coefficient, *β*_||_, was estimated to be as high as 0.99. As a result, the hindered diffusion was negligible.

### Determination of Brownian motion

Triple or dual color particle images were split using ImageJ by sampling hue range, which yielded image sets of each fluorescent color (Fig. 2C). A spatial cross-correlation algorithm was used to calculate the degree of Brownian motion of consecutive particle images in Matlab (Supplementary Fig. S1)^23^,^40^,^42^. Given a pair of images *I*_1_ and *I*_2_ at time *t* and *t* + Δ*t*, respectively, the displacement vector was calculated by a statistical method. Each image was divided into square-gridded regions called interrogation windows. By correlating two interrogation windows each time, a high correlation peak was obtained. The peak contains the information of a statistical displacement of overall particles in the interrogation window and the collective particle diffusion. In practice, fast Fourier transform is applied to calculate the image pairs for a correlation peak.

A Gaussian fit is used to outline the width and height of the correlation peak. Given that Brownian motion is usually regarded as random noise, the mean particle displacement is located near the center^40^,^42^. The width of the correlation peak is defined by 1/*e* intensity of the Gaussian distribution (*i.e., e* is the base of natural logarithm), which is derived from fitting the intensity profile with a two-dimensional Gaussian curve. The widths of the correlation peaks, ∆*S*_*a*_^2^ and ∆*S*_*c*_^2^, are derived from the auto-correlation and cross-correlation functions, respectively. The diffusivity, a statistical measure used to quantify Brownian motion, is expressed as


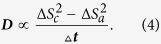


A broad correlation peak theoretically means strong diffusivity and vice versa. In our method, an increase in the width of the correlation function indicates the equivalent volume diameter of a particle attached to more bacteria^40^,^42^. Although an ideal correlation peak calculated from Brownian motion is expected to be located in the center of the correlation domain, a correlation bias is usually found dealing with motile bacteria, such as *P. aeruginosa*. On this basis, we sequentially rotated each correlation domain by 90° for a series of consecutive correlation peaks to achieve an ensemble average^23^. Thus, the bias and uncertainty were effectively reduced.

### Diffusivity of particles with equivalent volume diameter

Particles attached to bacteria formed irregular shape. For simplicity in analysis, a theoretical model equating the irregular particle into an ideal sphere was used to determine the diffusivity change resulting from the number of non-motile bacteria. In this model, the diameter of bacterium–particle complex was defined by the equivalent volume diameter as follows^30^:


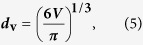


where *V* is the total volume of the attached bacteria and the carrier particle. Relative diffusivity values were then obtained by substituting *d*_V_ into Eq. (2). Experimental data were compared with those from predicted different particle sizes with different bacterial densities according to equivalent diameter (Eq. 5).

### Bacterial quantification by diffusometry

Bacteria were pretreated with 8 W 254 nm UV light (EBF-280 C, Spectroline^®^) at 4 °C for 24 h to obtain dead bacteria. Triple color particles were composed of equal amounts of anti-*P. aeruginosa*, anti-*S. aureus*, and anti-TNF-α Ab-functionalized particles. The anti-*P. aeruginosa* Ab- and anti-*S. aureus* Ab-functionalized particles were used to detect the bacteria simultaneously, and the anti-TNF-α Ab-functionalized particles served as a probe to reflect environmental conditions. Samples (~0.5 μL) containing particle–bacterium complexes were prepared for each measurement to verify the theoretical relationship between diffusivity and the complex. Data acquisition and image processing were performed after the particle–bacterium complexes were prepared. After 1 h incubation with different concentrations of mixed *P. aeruginosa* and *S. aureus*, particle images (in each group, image sets n = 10) were recorded every 0.1 s with a 10X objective for analyzing. The functionalized particles, *P. aeruginosa* and *S. aureus* were mixed at various ratios. The mean diffusivity values of each fluorescent particle in each group were obtained through cross-correlation algorithm. All measurements were finally divided by the diffusivity of anti-TNF-α Ab-functionalized particles to obtain relative values that were more suitable than absolute values because the variations in measurements result from different environmental factors or background noise.

### Antimicrobial susceptibility of co-cultured bacteria

A multiplexed AST was conducted by simultaneously monitoring the growth of co-cultured *P. aeruginosa* and *S. aureus* in TSB without or with gentamicin (0.2, 0.4, and 2 μg/ml). Gentamicin is an aminoglycoside antibiotic used in bacterial treatments The initial concentration of both bacteria, *P. aeruginosa* and *S. aureus*, was 10^5^ CFU/mL according to the Clinical and Laboratory Standards Institute. The ratio of the bacteria and their corresponding Ab-functionalized particles was 1:1. After incubation for 1 h, bacterial-binding particles were mixed with gentamicin in the TSB medium, and a small liquid droplet was then loaded into the PMMA chip at 37 °C. For each AST measurement, the chip was flipped over every 2 min, and the recording was performed every 20 min with a 10X objective in a total timespan of 2 h.

### Statistical analysis

Data from more than three independent experiments were presented as mean ± standard deviation. One-way ANOVA was used to analyze the data, and p value less than 0.05 was considered statistically significant.

## Additional Information

**How to cite this article:** Chung, C.-Y. *et al*. Simultaneous and quantitative monitoring of co-cultured *Pseudomonas aeruginosa* and *Staphylococcus aureus* with antibiotics on a diffusometric platform. *Sci. Rep.* **7**, 46336; doi: 10.1038/srep46336 (2017).

**Publisher's note:** Springer Nature remains neutral with regard to jurisdictional claims in published maps and institutional affiliations.

## Supplementary Material

Supplementary Data

## Figures and Tables

**Figure 1:**
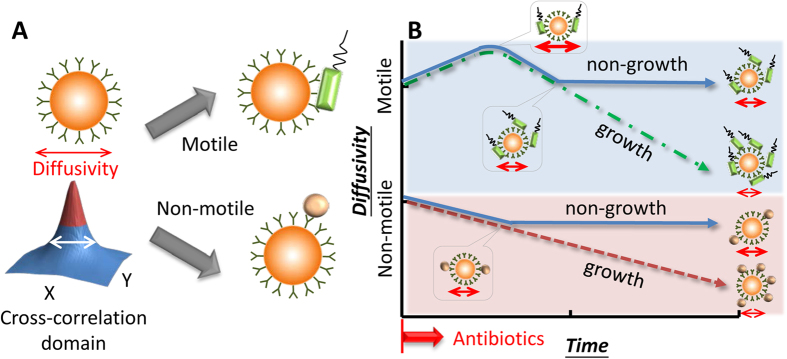
Conceptual illustration of the diffusometry-enabled technique for AST. (**A**) Fluorescent particles are measured by diffusometry and analyzed with the spatial cross-correlation algorithm for the information of diffusivity. Theoretically, a broad correlation width indicates strong diffusivity. The target microorganisms are captured based on immunoreactions. Motile or non-motile bacteria can lead to different diffusivity changes. (**B**) In the AST tests, motile bacteria, such as *P. aeruginosa*, boost the diffusivity initially owing to the bacterial motility. After more bacteria attach to particles, the diffusivity drops all the way down if the bacteria are resistant to antibiotics; otherwise, the diffusivity stops declining at a certain time point. By contrast, non-motile bacteria have no increased diffusivity along time. Apart from this difference, their other responses to antibiotics are similar to their motile counterpart.

**Table 1:** Binding efficacy and specificity of particles with different surface modifications attached to *P. aeruginosa* and *S. aureus*.

Particles modification	Particles attached with bacteria (%)					
	Amine	None	Carboxylate	Anti- *P. aeruginosa*	Anti-*S. aureus*	Anti-TNF-α
*P. aeruginosa*	41.0 ± 8.4	27.4 ± 5.5	0.0 ± 0.0	92.0 ± 2.2	0.0 ± 0.0	0.0 ± 0.0
*S. aureus*	24.8 ± 2.9	99.4 ± 0.4	100.0 ± 0.0	0.0 ± 0.0	85.4 ± 3.8	0.0 ± 0.0

**Table 2:** Ratios of bacteria to their corresponding particles*.

		*S. aureus*					
Ratios		0	10^−3^	10^−2^	10^−1^	10^0^	10^1^
*P. aeruginosa*	0	○				○	
	10^−3^			○			
	10^−2^		○	○	○		
	10^−1^			○	○		
	10^0^	○				○	○
	10^1^					○	○

*Triple color particles composed of equal amounts of anti-*P. aeruginosa* Ab- (orange), anti-*S. aureus* Ab- (red), and anti-TNF-α (green) Ab-functionalized particles (1:1:1).
