# Supplementary material for: Simultaneous and quantitative monitoring of co-cultured Pseudomonas aeruginosa and Staphylococcus aureus with antibiotics on a diffusometric platform
Source: Sci Rep. 2017 Apr 12;7:46336. doi: 10.1038/srep46336 (PMC5389350; doi:10.1038/srep46336)
Supplement: Supplementary Data [file srep46336-s1.pdf]

Simultaneous and quantitative monitoring of co-cultured *Pseudomonas aeruginosa* and *Staphylococcus aureus* with antibiotics on a diffusometric platform

Chih-Yao Chung[1], Jhih-Cheng Wang[2], Han-Sheng Chuang[1, 3, *]
[1]Department of Biomedical Engineering, National Cheng Kung University, Tainan, Taiwan.
[2]Department of Urology, Chimei Medical Center, Tainan, Taiwan.
[3]Medical Device Innovation Center, National Cheng Kung University, Tainan, Taiwan.
* oswaldchuang@mail.ncku.edu.tw

Derivation of diffusivity from particle images

The particle images were analyzed by spatially cross-correlation algorithm applied in Matlab (Fig. S1). An ideal correlation peak should locate in the center of the correlation domain because of the stationary colloidal suspension. Considering the motility *P. aeruginosa*, however, each of correlation peaks extracted from sequential image pairs was rotated 90° to previous one to offset the influence of microorganisms' activity. At last, the correlation peaks of the entire sequential image pairs set were ensemble and averaged in the correlation domains to reduce the bias and uncertainties. The width of an ensemble correlation peak was determined by delineating the intensity profile with a two dimensional Gaussian curve fit and defined by 1/*e* intensity of the Gaussian distribution (*i.e.*, *e* is the base of natural logarithm).

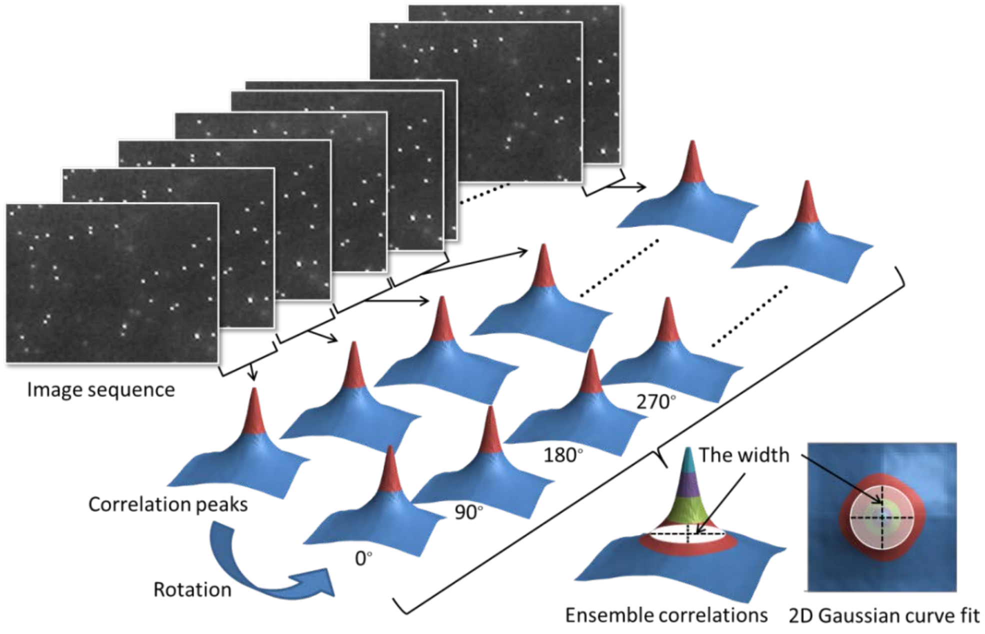

Fig. S1. Conceptual diagram of the computational procedure of the cross-correlation algorithm for a series of particle images.

Preparation of Ab-functionalized particles

Carboxylate -modified polystyrene beads (d_p=2 μm; L4530, L9529, or L3030; Sigma-Aldrich) were washed with MES buffer (pH 5.5) to prevent the aggregation. The particles were activated with 10 mg/ml EDC and 10 mg/ml NHS for 15 min. The EDC/NHS-activated particles were then incubated with 4 μl of polyclonal antibodies (ab67905, ab20920, or ab9348; Abcam) at 4 °C and 800 rpm for 4 h, and the final volumetric concentration was 0.625% v/v (Fig. S1).

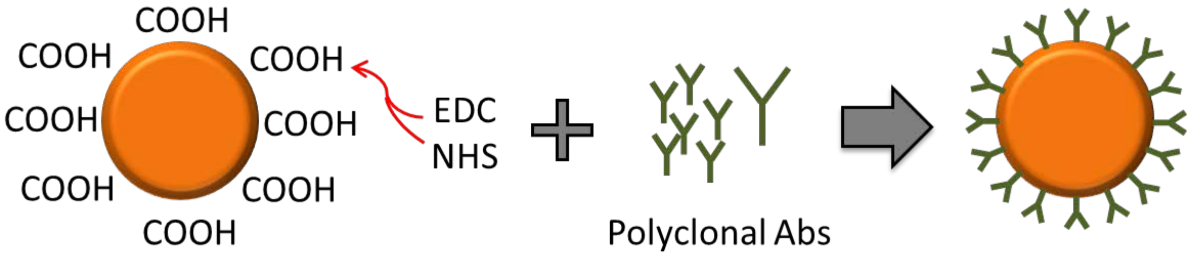

Fig. S1. Schematic of the particle functionalization.

Simultaneous detection with dual color particles

Green fluorescent particles were coated with anti-TNF-α Ab and served as a reference probe and orange fluorescent particles coated with anti-*P. aeruginosa* Ab to capture *P. aeruginosa*. The bright field image provided visual evidence showing the successful binding between the mixed bacteria and their corresponding particles (green: environmental reference; orange: *P. aeruginosa*) (Fig. S3). This result proved that simultaneous detection of multipathogens could be achieved by immunoassays.

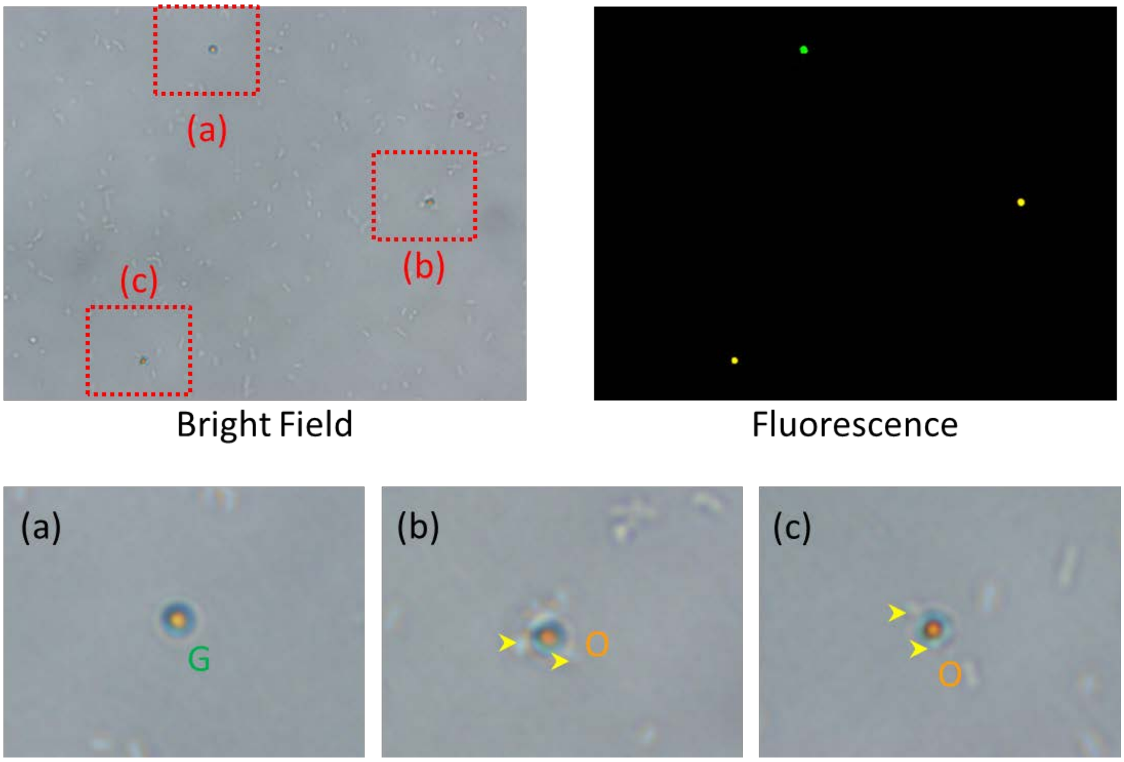

Fig. S3. Simultaneous detection with dual color particles (upper panel: bright field, left; fluorescent field, right). Close views (lower panel) of red rectangles labeled as (a), (b), and (c) in upper panel. (a) Anti-TNF-α Ab-functionalized green fluorescent particles free from bacterium binding (G in green). (b) and (c) *P. aeruginosa* attached to anti-*P. aeruginosa* Ab-functionalized orange fluorescent particles (O in orange).

Bacterial quantification with mono-colored particles

The UV-sterilized *P. aeruginosa* was incubated with the Ab-functionalized particles. The diffusivity of particles decreased monotonically according to the number of dead bacteria they were attached to (Fig. S4A). A similar phenomenon was observed in particles bound with nonmotile bacteria, *S. aureus*, in that a high number of bacteria always resulted in low diffusivity (Fig. S4B). Overall, the experimental data showed favorable agreements with the predicted curves of 2-μm particles (Figs S4A and B, the blue and red solid lines).

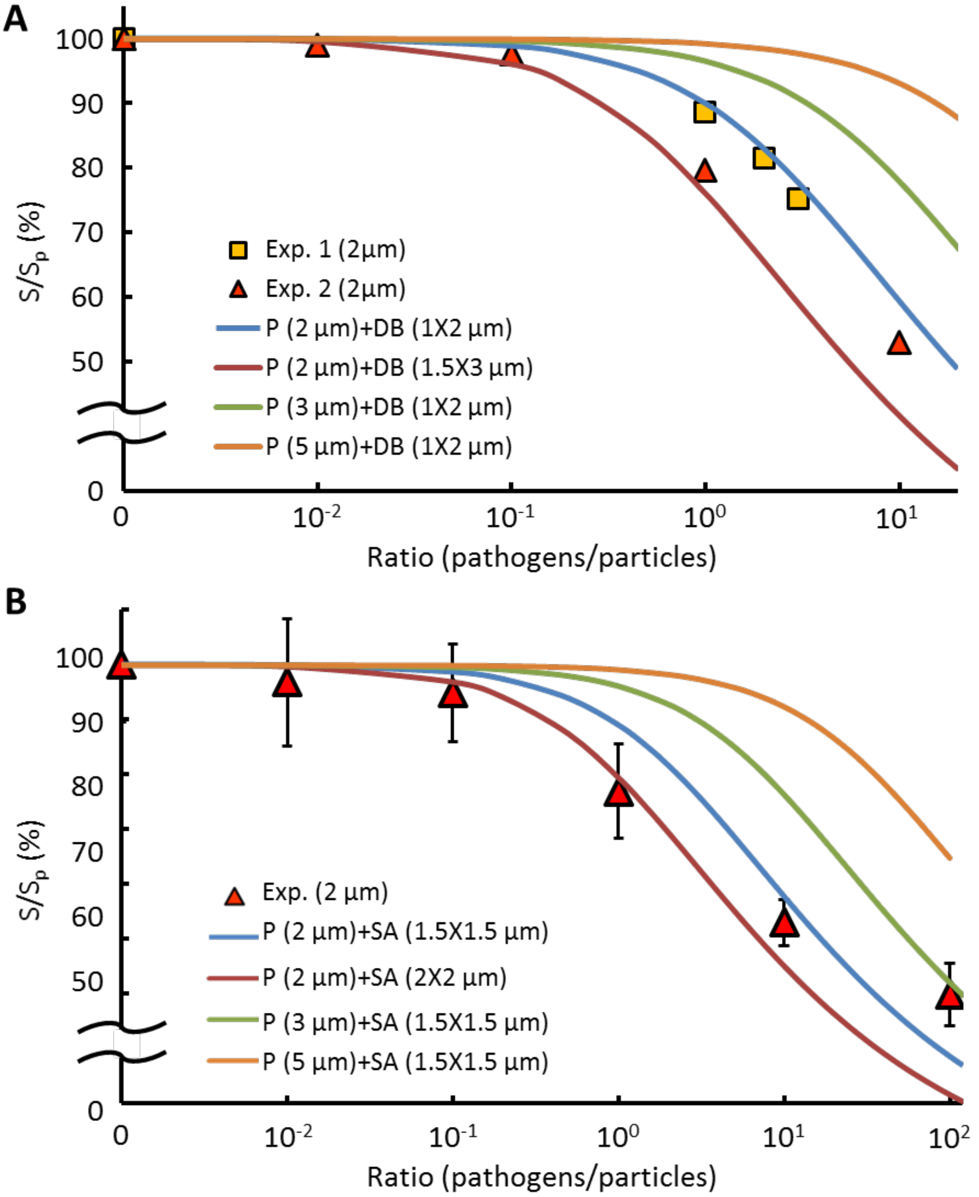

Fig. S4. Bacterial quantification with functionalized mono-colored particles. (A) Mean diffusivity values of anti-*P. aeruginosa* Ab functionalized particles attached with *P. aeruginosa* at different concentrations. (B) Mean diffusivity values of anti-*S. aureus* Ab functionalized particles attached with *S. aureus* at different concentrations. Solid lines are the predicted curves of different size particles with respect to different bacterial concentrations and sizes according to equivalent diameter theory. P means particles, DB means UV-sterilized *P. aeruginosa*, SA means *S. aureus*, S is the slope of regression line of particles with bacteria, and S_p is the slope of regression line of particles without bacteria.

Bacterial quantification with dual color particles

With dual color particles that were composed of equal amounts of anti-*S. aureus* and anti-TNF-α Ab-functionalized particles, the diffusivity values of the two functionalized particles at different *S. aureus* concentrations were recorded simultaneously. The diffusivity values of anti-*S. aureus* Ab-functionalized particles divided by those of anti-TNF-α Ab-functionalized particles were compared using the equivalent diameter theoretical model. The results showed favorable agreement with the predicted curves of 2-μm particles (Fig. S5).

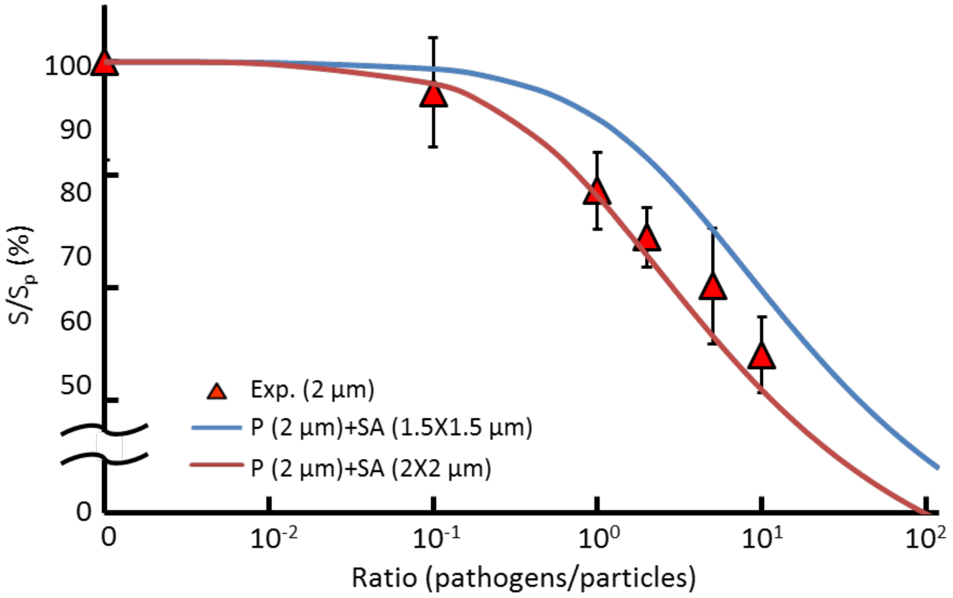

Fig. S5. Bacterial quantification with functionalized dual color particles. Mean diffusivity values of anti-*S. aureus* Ab functionalized particles with *S. aureus* at different concentrations were divided by those of anti-TNF-α Ab-functionalized particles. The divided diffusivity values were compared using the equivalent diameter theoretical model. Solid lines are the predicted curves of 2-μm different size particles with respect to different bacterial concentrations and sizes according to equivalent diameter theory. P means particles, SA means *S. aureus*, S is the slope of regression line of particles with bacteria, and S_p is the slope of regression line of particles without bacteria.

Preventing sedimentation by flipping for continuous monitoring

Although the density of particles was close to water (ρ = 1.05 g/cm^3), sedimentation could still disturb its diffusivity when the particles were near the chip walls. For continuously monitoring the growth of polymicrobial infection pathogens over a long timescale (at least 2 h), sample droplets loaded in chips coated with or without BSA were flipped at rate of 0, 1, and 2 min^{-1} over 2 h. Numbers of particles at middle and near-wall plates were calculated. Results showed that flipping a chip with BSA coating at rate of 1 and 2 min^{-1} could effectively avoid particle sedimentation on the near-wall plate (Fig. S6).

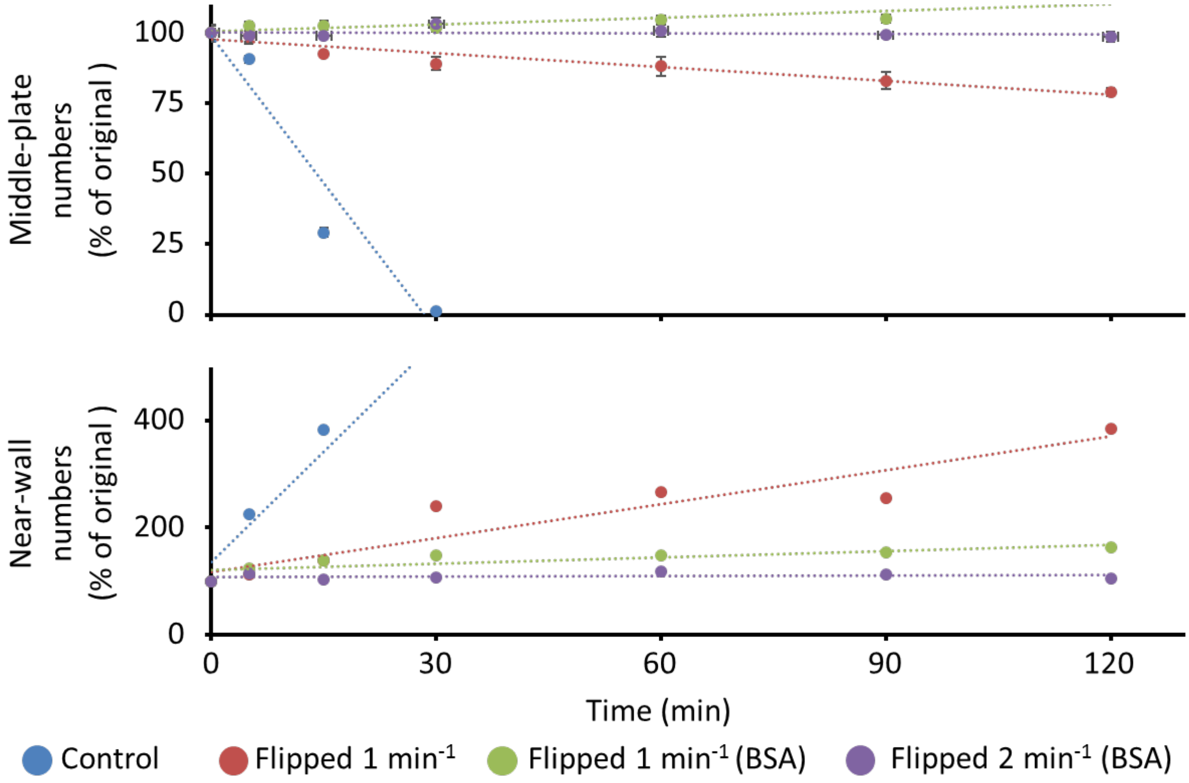

Fig. S6. Flipping prevents sedimentation to achieve continuous monitoring. Chips coated with or without BSA were flipped at rate of 0, 1, and 2 min-1 over 2 h. Numbers of particles at middle (upper panel) and near-wall plates (lower panel) were calculated.

Rapid antimicrobial susceptibility testing by diffusometry

An AST process was assessed by measuring the growth of *P. aeruginosa* and *S. aureus* in TSB with antibiotics. The initial density of *P. aeruginosa* and *S. aureus* was 10^5 CFU/mL according to the guideline of Clinical and Laboratory Standards Institute. The density ratio of the bacteria and the Ab-functionalized particles was maintained at 1:1. After 1 h of incubation, the bacteria were respectively mixed with antibiotics at different concentrations in the TSB medium at 37 °C and 800 rpm for 2 h. Particles images were recorded every 20 min with a 20× objective to monitor the bacterial activity.

In the *P. aeruginosa* mono-cultured condition, the diffusivity changes of the particles in

the control group and in the group of 0.02 μg/mL gentamicin all exhibit increases in the first 20 min, followed by constant decreases. By contrast, the diffusivity changes of the particles in the presence of 0.5 and 2 μg/mL gentamicin exhibit slight decreases in the first 60 min and then show no change afterward (Fig. S7A).

In the *S. aureus* mono-cultured condition, the diffusivity changes in the functionalized particles in the control group and in the group of 0.1 μg/mL cefapime only displayed a constant decline, whereas the diffusivity of the particles in the presence of 1 and 4 μg/mL cefapime did not change (Fig. S7B).

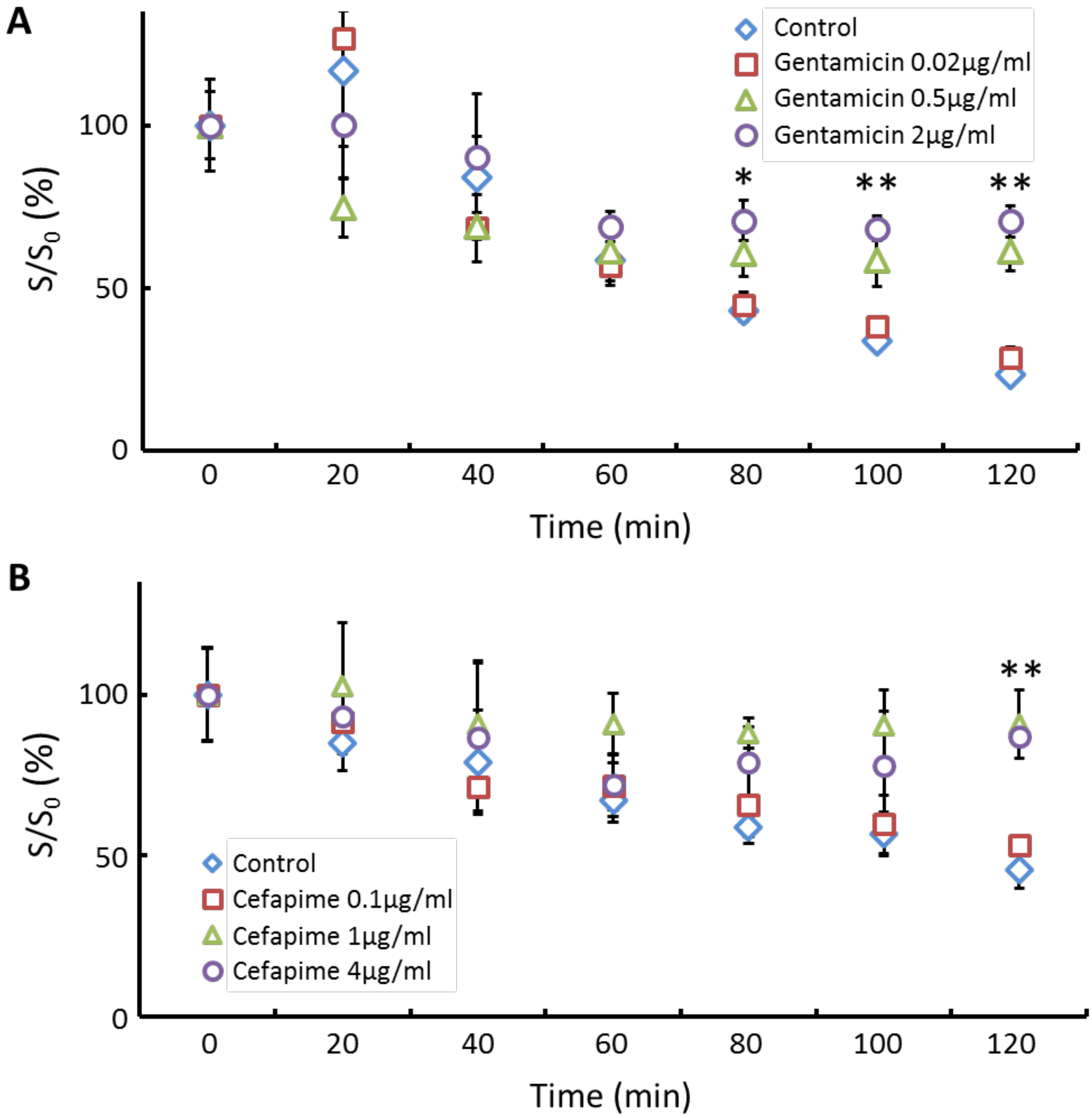

Fig. S7. Rapid antimicrobial susceptibility testing by diffusometry. (A) Temporal diffusivity changes of particles attached with *P. aeruginosa* in the presence or absence of gentamicin (0.02, 0.5, and 2 μg/mL). (B) Temporal diffusivity changes of particles attached with *S. aureus* in the presence or absence of cefapime (0.1, 1, and 4 μg/mL). S is the slope of regression lines and S_0 is the regression line slope of particles measured at 0 min. * $p < 0.05$, and ** $p < 0.01$.

Antimicrobial susceptibility testing by broth microdilution

Conventional AST was conducted to measure the growth of co-cultured *P. aeruginosa* and *S. aureus* (10^5 CFU/mL each) in TSB with 0, 0.2, 0.4, and 2 μg/mL gentamicin, according to the guideline of Clinical and Laboratory Standards Institute. Briefly, 100 μL mixed bacteria suspension was distributed to the 96-well plate. The mixed bacteria was incubated with 0, 0.2, 0.4, and 2 μg/mL gentamicin at 37°C for 48 h. The turbidity of each groups was determined by using a plate reader (530 nm) at 24 h and 48 h.

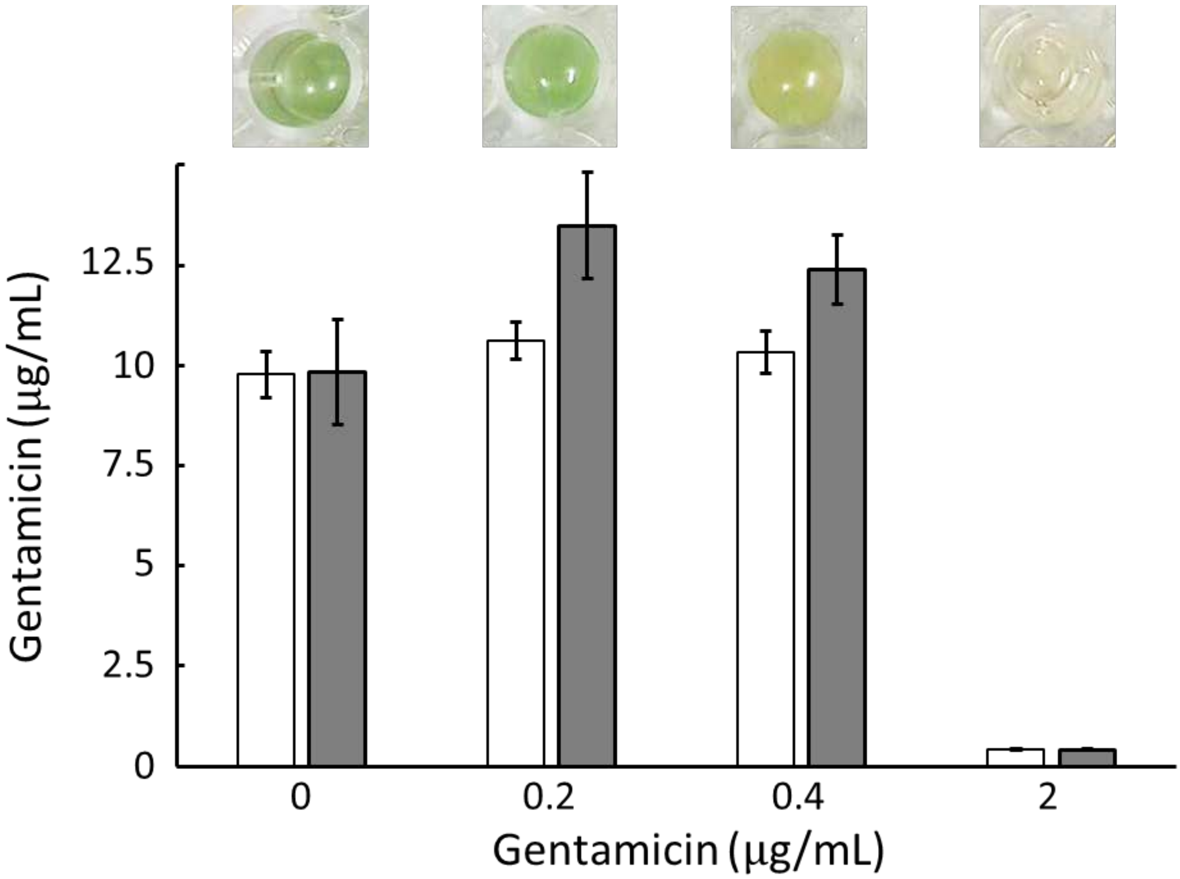

Fig. S8. Broth microdilution. Co-cultured *P. aeruginosa* and *S. aureus* (10^5 CFU/mL each) in TSB with 0, 0.2, 0.4, and 2 μg/mL gentamicin were incubated at 37°C for 48 h. A plate reader (530 nm) was applied to determine the turbidity of each groups at 24 h and 48 h.
